# Clinicopathological Diagnosis and Prognosis of Endometrioid Borderline Ovarian Tumors: Dual Case Reports and Literature Review

**DOI:** 10.1002/cnr2.70388

**Published:** 2025-10-28

**Authors:** Fang Yang, Dong Chen, Qian Sun, Jingjing Yu, Xue Wang

**Affiliations:** ^1^ Department of Pathology Ningbo Urology and Nephrology Hospital, Ningbo Yinzhou No. 2 Hospital Ningbo Zhejiang China; ^2^ Department of Obstetrics and Gynecology Ningbo Urology and Nephrology Hospital, Ningbo Yinzhou No. 2 Hospital Ningbo Zhejiang China; ^3^ Urology and Nephrology Institute Ningbo Urology and Nephrology Hospital, Ningbo Yinzhou No. 2 Hospital Ningbo Zhejiang China

**Keywords:** case report, *CTNNB1*, endometrioid borderline ovarian tumors, prognosis, *PTEN*

## Abstract

**Introduction:**

Endometrioid borderline ovarian tumor (EBOT) is rare and frequently misdiagnosed. This study aims to investigate the clinicopathological features, immunohistochemical characteristics, differential diagnosis, therapeutic approaches and disease prognosis, thereby establishing a robust foundation to mitigate misdiagnosis risks and deepen insights into the pathological diagnosis of this disease.

**Case Presentations:**

From May 2020 to December 2022, two female patients diagnosed with EBOT were enrolled at Ningbo Yinzhou No. 2 Hospital. The patients, aged 30 and 34 years, respectively, both underwent left adnexal resection. Microscopically, the tumors displayed disorganized crowded endometrioid glands, mild‐to‐moderate epithelial atypia, and fibrous stroma interspersed among glands. Mulberry‐like squamous metaplasia was also observed in some areas. Tumor cells were positive for cytokeratin (CK), cytokeratin 7 (CK7), estrogen receptor (ER) and progesterone receptor (PR), but negative for Wilms' tumor 1 (WT‐1). The Ki‐67 index ranged from 3% to 10%. Genetic analysis revealed a heterozygous *CTNNB1* deletion in one tumor, whereas a heterozygous *PTEN* deletion in the other. As of the current follow‐up (ranging from 10 to 40 months), both cases remained in a tumor‐free status, with no signs of recurrence or metastasis to date.

**Conclusion:**

EBOT are infrequent and may coexist with endometriosis or endometrioid carcinoma. Our cases demonstrated a heterozygous deletion of the *CTNNB1* gene in one case, while a heterozygous deletion of the *PTEN* gene in the other. Surgery remains the main treatment, reflecting its efficacy in achieving disease control and a favorable prognosis.

## 1 Introduction

In the fifth edition of the World Health Organization (WHO) classification of Female Genital Tumours, endometrioid ovarian tumors are categorized into three types: benign, borderline, and malignant, encompassing endometrioid cystadenoma, endometrioid cystadenofibroma, endometrioid borderline ovarian tumor (EBOT), and endometrioid carcinoma [1, 2, 3, 4, 5, 6]. Compared to serous and mucous ovarian tumors, EBOT occurs far less frequently, accounting for approximately 0.2% of all epithelial ovarian tumors [1, 2, 3, 4, 5, 6]. These tumors are characterized by densely packed endometrioid glands with atypical epithelium, lacking fusion or destructive stromal invasion. Currently, clinical and pathological understanding of EBOT remains limited, with sparse literature addressing their prognostic factors, therapeutic strategies, and molecular mechanisms of development.

This study retrospectively analyzed the clinical and pathological characteristics of two EBOT cases from Yinzhou No. 2 Hospital, aiming to enhance clinicians' and pathologists' understanding of the disease, facilitate accurate diagnosis, and prevent misdiagnosis or inappropriate treatment.

## 2 Case Description

Data from two EBOT patients were obtained from the pathology archives of Ningbo Yinzhou No. 2 Hospital between May 2020 and December 2022. Two pathologists reviewed the cases and reached a diagnostic consensus. Clinical data, imaging materials, gross pathological features, and follow‐up information were collected through the hospital's electronic medical record system. The study was approved by the Ethics Committee of Ningbo Yinzhou No. 2 Hospital (2023‐P‐044) and was conducted according to the Declaration of Helsinki.

Patient 1 was a 34‐year‐old female. Ultrasonography revealed a solid‐cystic lesion in the left adnexa measuring 103 mm × 60 mm, while computed tomography (CT) imaging showed a larger mass in the same area, quantified at 106 mm × 65 mm. Serological assessments demonstrated an elevated serumcarbohydrate antigen 125 (CA125) level of 51.4 U/mL (normal range: 0–35 U/mL).

**FIGURE 1:**
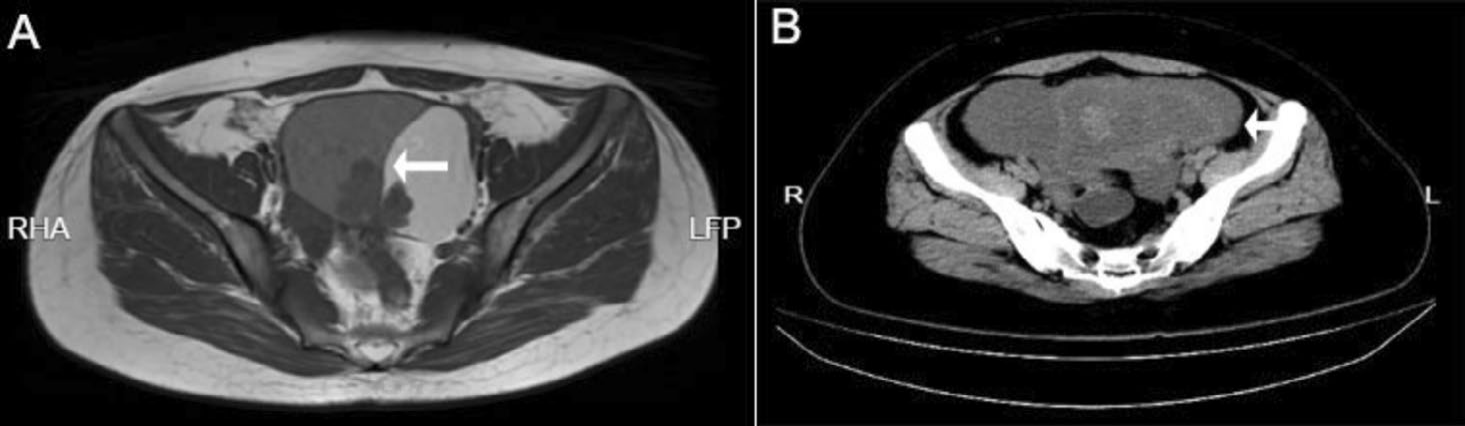
Preoperative CT results of two cases. (A) In case 1, CT imaging revealed a large cystic solid mass in the left adnexal region, measuring 106 × 65 mm, with a solid component resembling cabbage measuring 47 × 32 mm. (B) In case 2, there were short T1 and long T2 signals, presenting as a 46 × 91 mm flaky mass with a thick wall in the left adnexal area. The mass had clear edges, with a shadow measuring 25 × 29 mm.

While, patient 2 was a 30‐year‐old female, admitted to the hospital due to unbearable periumbilical pain. Ultrasonography examination identified a solid‐cystic lesion in the left adnexa measuring 47 mm × 30 mm. CT imaging showed pelvic hematoma and an abnormal mass in the left adnexal region measuring approximately 25 mm × 29 mm (Figure 1B). Serological assessments indicated both an abnormal CA125 level of 3268.7 U/mL (range: 0–35 U/mL) and a carbohydrate antigen 199 (CA199) level of 47.8 U/mL (range: 0–25 U/mL).

Two patients both underwent left adnexectomy, and postoperative specimens were submitted for histopathological evaluation. They recovered well post‐surgery, with no recurrence observed during follow‐up.

## 3 Methods

### 3.1 Hematoxylin and Eosin and Immunohistochemistry Staining

All specimens were fixed in 10% neutral formalin and embedded in paraffin. Conventional hematoxylin and eosin (H&E) staining was performed, followed by immunohistochemical (IHC) staining using the EnVision two‐step method according to the manufacturer's instructions. The primary antibodies utilized included broad‐spectrum cytokeratin (CKpan), cytokeratin 7 (CK7), cyclin‐dependent protein kinase 4 (P16), mismatch repair proteins (MLH1, PMS2, MSH2, MSH6), P63, Wilms' Tumor 1 (WT‐1) (purchased from Zhongshan Jinqiao Co. Ltd.), as well as the estrogen receptor (ER) and progesterone receptor (PR) from Roche. All the antibodies above are readymade. Results Interpretation: Positive immunohistochemical staining was identified by a brownish‐yellow or brown color, while the absence of staining was considered negative. ER, PR, P63, ki67, MLH1, PMS2, MSH2, and MSH6 showed positive staining located in the nucleus, whereas CKpan, CK7, CK5/6, WT‐1, and P16 exhibited positive staining in the cell membrane and cytoplasm.

### 3.2 Fluorescence in Situ Hybridization

Gene probes and the fluorescence in situ hybridization (FISH) kit were sourced from Guangzhou Ambipin Medical Technology Co. Ltd., and experimental procedures were conducted according to the kit instructions. Under a fluorescence microscope, nuclear fluorescence signals of 100 tumor cells were randomly counted. Heterozygous deletion of *PTEN* and *CTNNB1* was defined as the absence of one red fluorescence signal in more than 20% of tumor nuclei, while homozygous deletion was characterized by the absence of two red fluorescence signals in more than 30% of tumor nuclei. *PIK3CA* gene amplification was indicated by a ratio of red to green fluorescence signals greater than 2 (based on 40 randomly counted cells).

## 4 Results

### 4.1 Histologic Characteristics

**FIGURE 2:**
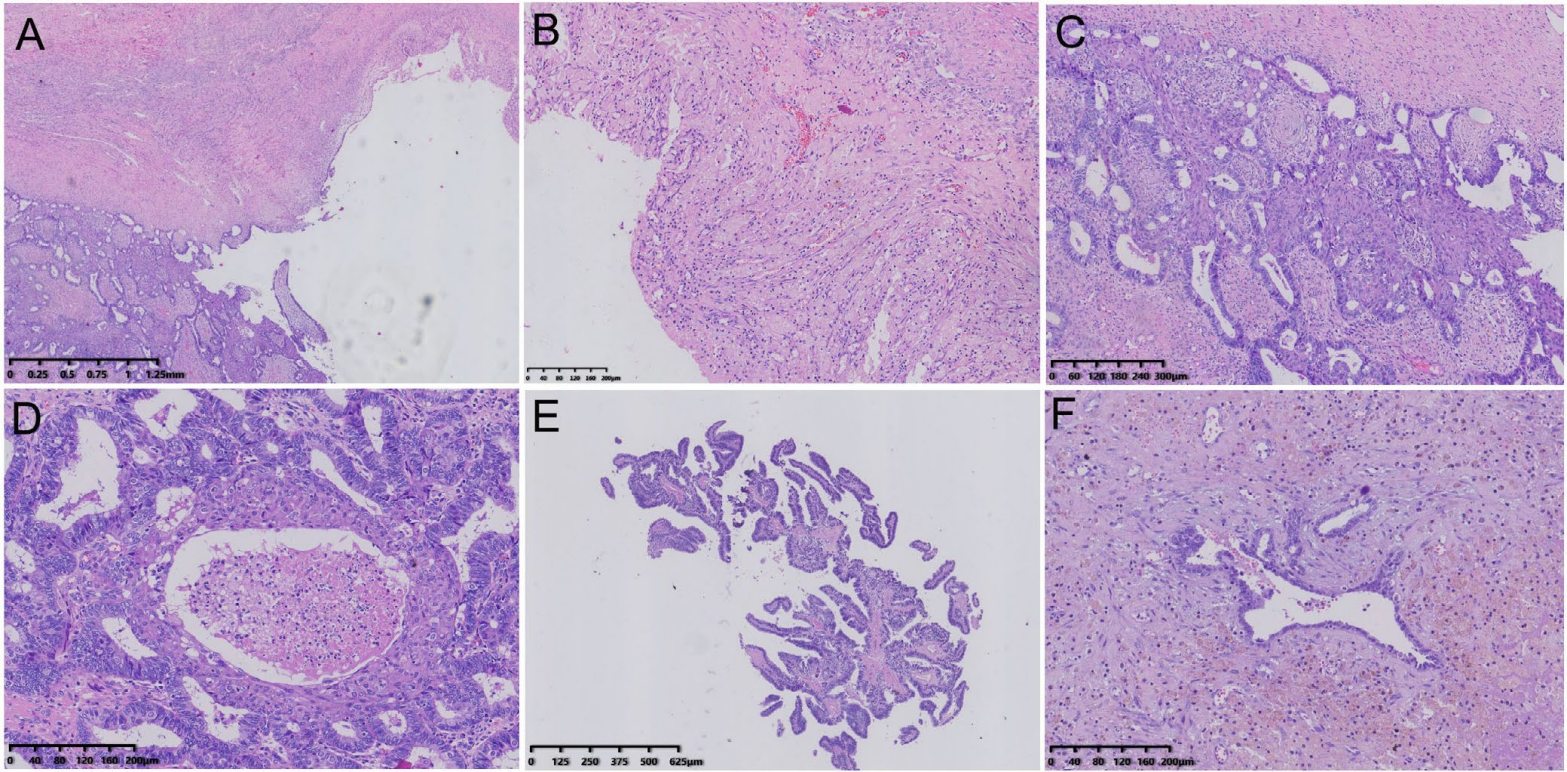
Histopathological morphology of EBOT. (A) Cystic and focally solid area in case 1. (B) Luteinization in the wall of the cystic area. (C) Endometrioid glands in the solid area were fused back‐to‐back, with variable‐sized cavities and cells exhibiting mild to moderate atypia, accompanied by squamous differentiation. (D) Focal necrotic fragments. (E) In case 2, most of the area was cystic; the solid area was primarily papillary, with some endometrioid glands showing back‐to‐back fusion and squamous differentiation. (F) The endometriosis area.

Two patients both were admitted to our hospital in May 2020 and November 2022, respectively. Subsequently, CT examinations revealed ovarian occupancy in both (Figure 1A,B). Therefore, they underwent left adnexectomy on May 6, 2020 and November 23, 2022, respectively, and the surgically resected specimens were sent to the pathology department for gross and microscopic evaluation. Macroscopically, both tumors were predominantly cystic with focal solid areas (Figure 2A). Case 1 demonstrated luteinized cyst walls histologically (Figure 2B). Within its solid area, crowding of endometrioid glands formed contiguous back‐to‐back architectures with variable lumen sizes, displaying mild‐to‐moderate nuclear atypia and squamous metaplasia (Figure 2C), alongside foci of necrosis (Figure 2D). In case 2, the solid region was composed of a predominance of papillary structures with squamous differentiation (Figure 2E), as well as foci of endometriosis (Figure 2F). Therefore, the diagnosis of EBOT was confirmed in both of them.

### 4.2 Immunohistochemistry and Molecular Genetic Features

**FIGURE 3:**
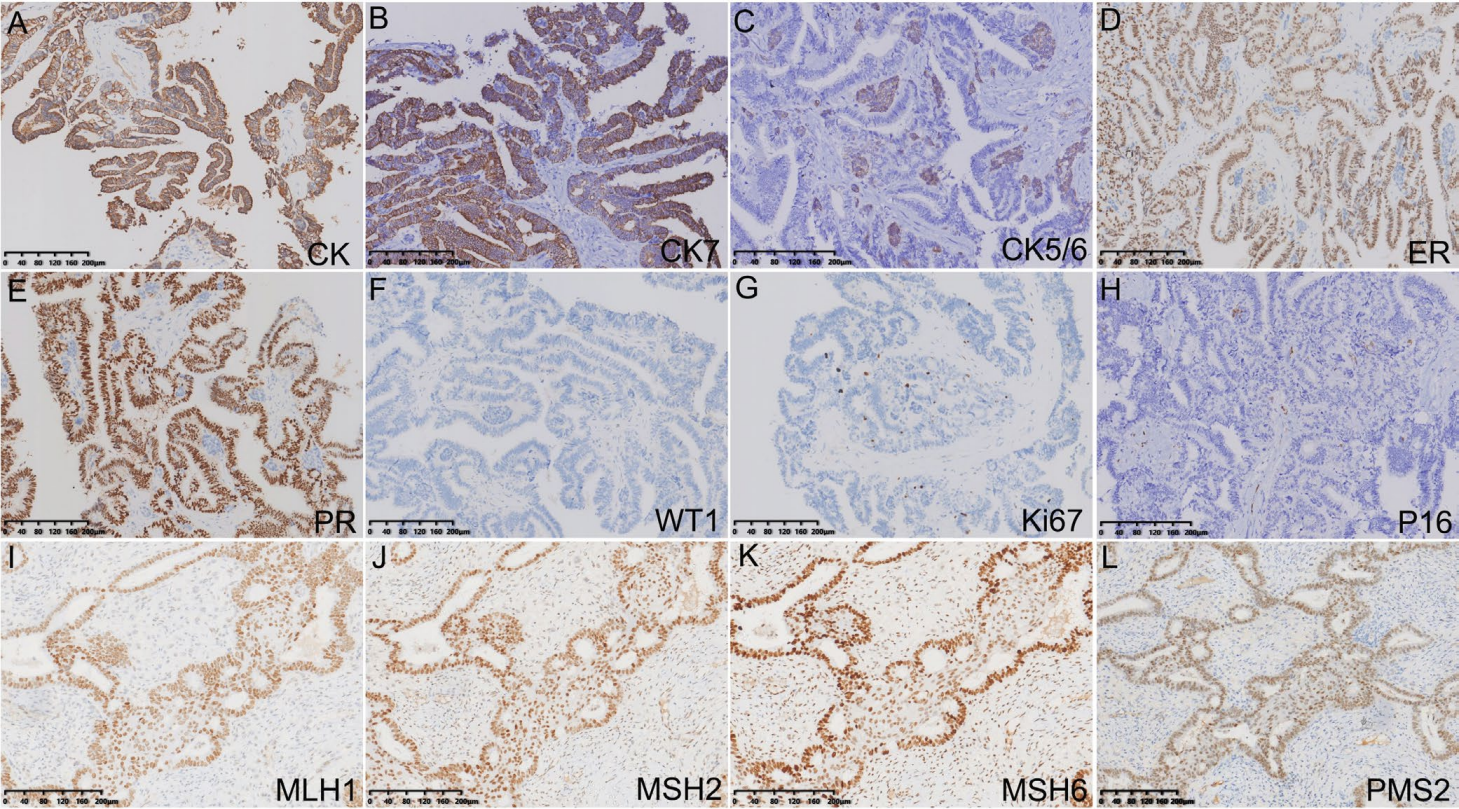
Immunohistochemical staining of EBOT. (A–H) Tumor cells showed strong positivity for CKpan, CK7, ER, and PR, while metaplastic squamous cells were positive for CK5/6. Tumor cells were negative for WT‐1, and the Ki‐67 proliferation index was 3%. Interstitial cells displayed focal positivity for P16. (I–L) Tumor cells were positive for MLH1, MSH2, MSH6, PMS2 in case 2.

**FIGURE 4:**
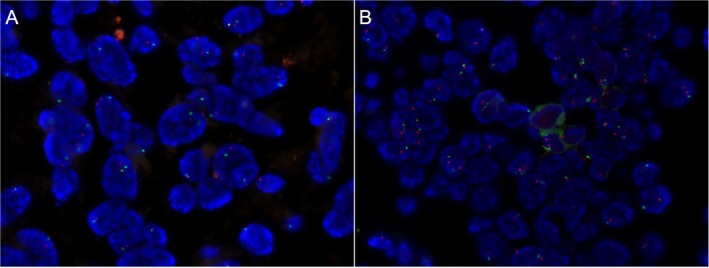
FISH results of two cases. (A) *CTNNB1* deletion in case 1, with a heterozygous deletion proportion of 32%. (B) *PTEN* deletion in case 2, with a heterozygous deletion proportion of 28%.

In both cases, the endometrioid glands showed strong expression of CKpan, CK7, ER, PR, MLH1, MSH2, MSH6, and PMS2 (Figure 3A,B,D,E,I–L), but negative WT‐1 (Figure 3F). Additionally, the squamous metaplastic cells were positive for CK5/6 (Figure 3C). The proliferation index of Ki‐67 in cases 1 and 2 was 10% (Figure S1) and 3% (Figure 3G), respectively. Case 1 presented a heterozygous deletion of *CTNNB1* (Figure 4A) while there was a heterozygous deletion of *PTEN* in another case (Figure 4B).

### 4.3 Treatment and Prognosis

Patient 1 underwent left adnexectomy on May 6, 2020, followed by a 3‐day course of intravenous cefoxitin sodium for postoperative infection prophylaxis. Hospitalization lasted 10 days post‐surgery, and the patient experienced an uneventful recovery with no complications during a 5‐year follow‐up period. Patient 2 underwent left adnexectomy on November 23, 2022. Postoperatively, she received intravenous infusion of antibiotics—cefoperazone sodium/sulbactam sodium—for 3 days and was discharged 6 days after surgery. Clinical follow‐up for 30 months confirmed no recurrence or complications in this patient.

## 5 Discussion

Ovarian borderline tumors, a subset of low‐grade epithelial neoplasms, comprise 10%–20% of ovarian epithelial tumors, while they typically exhibit a favorable prognosis with 80% of cases confined to the ovary [1, 2, 3, 4, 5, 6]. Borderline endometrioid tumors are exceptionally rare, representing approximately 0.2% of ovarian epithelial tumors, which are limitedly reported. Previous studies have indicated a disproportionately higher incidence in postmenopausal populations, where approximately 30% of cases occur in women younger than 40 years [3, 7, 8]. In contrast, our series exclusively involved two reproductive‐aged patients (30 and 34 years old). Histologically, borderline endometrioid tumors are distinguished by two predominant microscopic patterns: an adenofibromatous architecture and glandular/papillary formations. These tumors are characterized by crowded, back‐to‐back proliferative glands with mild to moderate atypia, frequent squamous (morular) metaplasia, and highly cellular or fibrous stroma. Severe cellular atypia warrants a diagnosis of intraepithelial carcinoma. Microinvasion is defined by a fusion area (stromal invasion) of less than 5 mm, while invasion exceeding 5 mm is classified as invasive carcinoma (endometrioid carcinoma). In case 1, the tumor demonstrated a solid‐predominant morphology with luteinized cyst walls. The solid areas revealed contiguous back‐to‐back endometrioid gland fusion, variable luminal dimensions, mild‐to‐moderate cytologic atypia, and squamous differentiation within the solid regions. Conversely, case 2 was cystic‐dominant, with solid components histologically characterized by papillary architectures and scattered endometrioid glands exhibiting back‐to‐back fusion and squamous metaplasia. Both neoplasms were histologically diagnosed as borderline endometrioid tumors originating from endometrioid cysts. The absence of uterine pathology in both patients strengthens the interpretation of primary ovarian tumor development.

Currently, it is believed that the mechanism of ovarian endometrioid tumors mainly originates from the surface epithelium of the ovary or the ectopic endometrium, with 15%–50% of patients exhibiting concurrent ovarian or extraovarian endometriosis. Our two cases were histologically associated with endometriosis cysts. Prior studies indicate significant comorbidity between endometriosis and EBOT: Zhang et al. identified endometriosis in 36.5% (*n* = 19/52) of EBOT cases, with affected patients displaying a younger age distribution compared to disease‐only cohorts [9]. In a cohort of 48 EBOT patients (median age, 52 years; 2022 study [10]), histologically confirmed endometriosis was present in 25% (*n* = 12 cases), and 24% (*n* = 7/29) of cases with recorded endometrial evaluations showed synchronous endometrial pathology [10]. These findings parallel our observations, as both cases demonstrated endometriosis cysts, suggesting that endometriosis may progress to borderline endometrioid tumors through an atypical stage and potentially further develop into endometrioid carcinoma.

Some studies have found that the major molecular alterations of EBOT include alterations of the *CTNNB1* gene (50%), and the *PTEN* gene (20%), as well as microsatellite instability (50%), which are also observed in uterine endometrioid carcinoma, supporting the hypothesis that endometriosis may be a precursor to EBOT. In a recent review study, through a detailed analysis of 15 molecular characteristics by Pawel Sadlecki et al., the genetic progression of borderline ovarian tumors towards low‐grade carcinoma via the ‘low‐grade pathway’ was attributed to mutations along the RAS/RAF/MEK/MARK pathway [11]. In our study, amplification/deletion of the *PTEN*, *CTNNB1*, and *PIK3CA* genes were found by FISH in two cases. Case 1 exhibited heterozygous loss of *CTNNB1*, while case 2 showed heterozygous loss of the *PTEN* gene, both consistent with described molecular profiles of EBOTs. Immunohistochemical assessment of mismatch repair proteins revealed no protein expression deficits, confirming the absence of microsatellite instability (MSI) in these cases.

The cornerstone of management is surgical resection, with guidelines from the National Comprehensive Cancer Network (NCCN) recommending hysterectomy in the absence of fertility preservation needs. For younger patients aiming to preserve fertility, endometrial assessment via curettage is advised to inform therapeutic decision‐making. Surgical extent is guided by patient age, fertility status, and disease staging. Analysis of a cohort study by Zhang et al. [9], which prospectively followed 52 EBOT patients, noted disease recurrence in nine cases but no mortality. Prognostic analysis identified favorable outcomes in patients aged over 50 years, in menopause, with serum CA125 levels below 140 U/mL, and tumor diameters under 10 cm. Our two cases, case 1 (aged 34) and case 2 (aged 30), who underwent left adnexal resection on May 6, 2020 and November 23, 2022 respectively, demonstrate an excellent prognosis, with no evidence of recurrence or metastasis detected to date.

Pure EBOT rarely shows malignant biological behaviors such as recurrence and metastasis, mostly associated with a favorable prognosis [8, 12]. During the follow‐up of 52 cases reported by Zhang et al. [9], disease progression or recurrence occurred in 9 cases, and no death was observed. However, endometrial biopsy results from EBOT patients frequently identify coexisting pathology, including endometrial polyps, hyperplasia, atypical hyperplasia, or malignancy. In the cases reported by Roth, 18 underwent endometrial evaluation, revealing proliferative changes in 2 cases and endometrial carcinoma in 5 [8]. In our cohort, endometrial biopsy of case 1 demonstrated hyperplastic changes, while case 2 lacked such evaluation, precluding confirmatory histopathologic correlation. Therefore, these findings highlight the clinical necessity for standardized endometrial assessment via biopsy.

## 6 Conclusion

Despite the favorable prognosis associated with EBOTs, their low incidence and nonspecific clinical features often result in diagnostic challenges. Our cases indicate the necessity of preserving reproductive function in surgically treated childbearing‐age patients. Notably, the risk of misdiagnosis as endometrioid carcinoma remains significant, necessitating enhanced diagnostic precision in pathological evaluation to avoid incorrect categorization and prevent unnecessary interventions such as overtreatment through aggressive surgical or adjuvant therapies.

## Author Contributions

Conceptualization: Jingjing Yu and Xue Wang. Methodology: Qian Sun and Jingjing Yu. Validation: Fang Yang, Dong Chen and Jingjing Yu. Formal analysis: Dong Chen. Data curation: Fang Yang, Dong Chen and Qian Sun. Writing – original draft preparation: Fang Yang. Writing – review and editing: Xue Wang. Visualization: Qian Sun. Project administration: Xue Wang. Funding acquisition: Xue Wang, Jingjing Yu and Fang Yang. All authors have read and agreed to the published version of the manuscript.

## Ethics Statement

The studies involving humans were approved by the Ethics Committee of Ningbo Yinzhou No. 2 Hospital (2023‐P‐044), in accordance with local regulations and institutional policies. Written informed consent was obtained from the patient(s) for both participation in the study and publication of this case report.

## Conflicts of Interest

The authors declare no conflicts of interest.

## Supporting information


**FIGURE S1:** Immunohistochemical staining of EBOT. The Ki‐67 proliferation index was 10% in case 1.

## Data Availability

The data that support the findings of this study are available from the corresponding author upon reasonable request.
