# Supplementary figures and images for: Clinicopathological Diagnosis and Prognosis of Endometrioid Borderline Ovarian Tumors: Dual Case Reports and Literature Review
Source: Cancer Rep (Hoboken). 2025 Oct 28;8(11):e70388. doi: 10.1002/cnr2.70388 (PMC12568370; doi:10.1002/cnr2.70388)

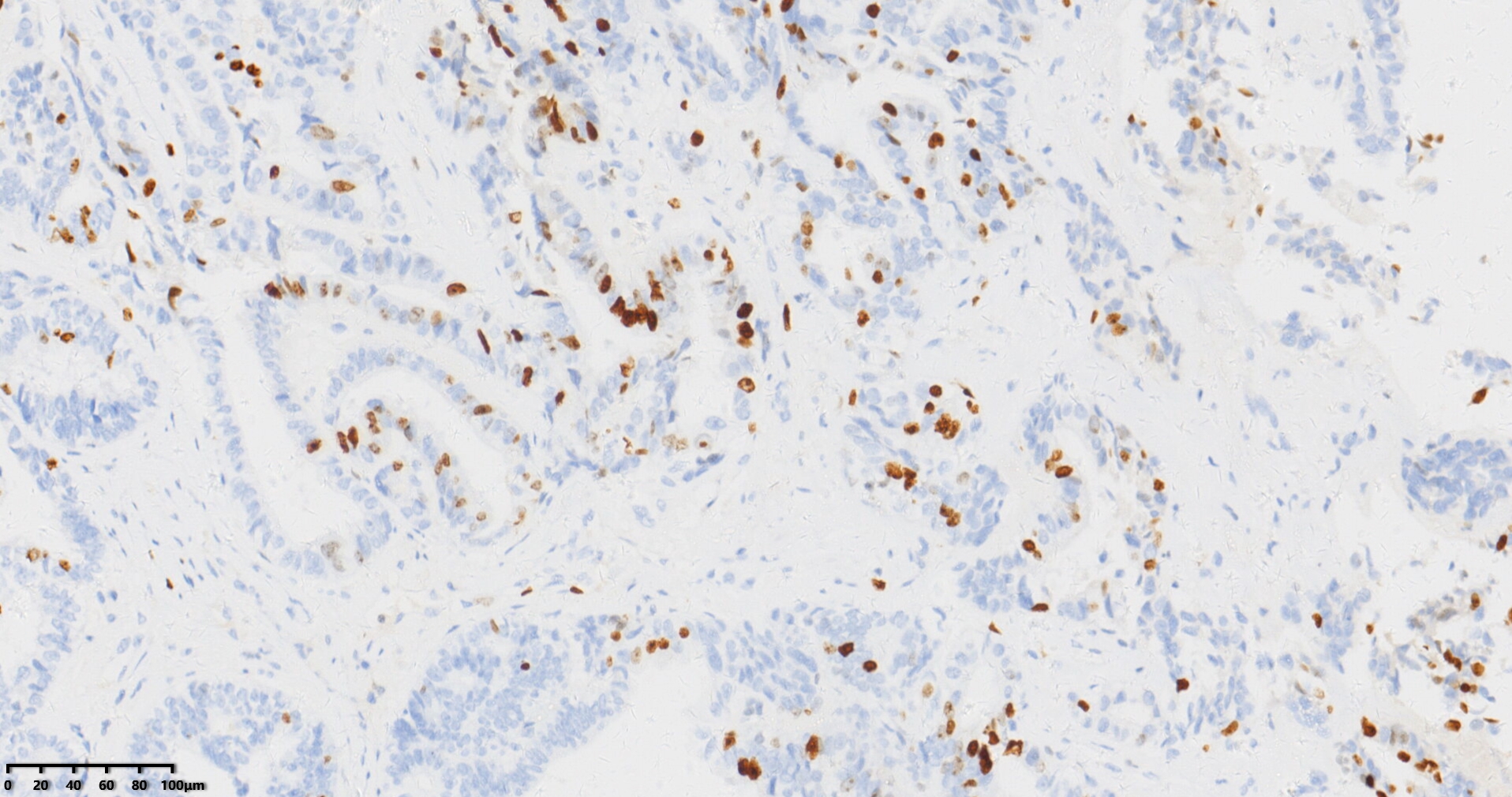

Supplement: Supplementary file 1 — FIGURE S1: Immunohistochemical staining of EBOT. The Ki‐67 proliferation index was 10% in case 1. [file CNR2-8-e70388-s001.jpg]
